# Are Famine Food Plants Also Ethnomedicinal Plants? An Ethnomedicinal Appraisal of Famine Food Plants of Two Districts of Bangladesh

**DOI:** 10.1155/2014/741712

**Published:** 2014-02-20

**Authors:** Fardous Mohammad Safiul Azam, Anup Biswas, Abdul Mannan, Nusrat Anik Afsana, Rownak Jahan, Mohammed Rahmatullah

**Affiliations:** Department of Biotechnology & Genetic Engineering, Faculty of Life Sciences, University of Development Alternative, House No. 78, Road No. 11A (new), Dhanmondi, Dhaka 1209, Bangladesh

## Abstract

Plants have served as sources of food and medicines for human beings since their advent. During famines or conditions of food scarcity, people throughout the world depend on unconventional plant items to satiate their hunger and meet their nutritional needs. Malnourished people often suffer from various diseases, much more than people eating a balanced diet. We are hypothesizing that the unconventional food plants that people eat during times of scarcity of their normal diet are also medicinal plants and thus can play a role in satiating hunger, meeting nutritional needs, and serving therapeutic purposes. Towards testing our hypothesis, surveys were carried out among the low income people of four villages in Lalmonirhat and Nilphamari districts of Bangladesh. People and particularly the low income people of these two districts suffer each year from a seasonal famine known as Monga. Over 200 informants from 167 households in the villages were interviewed with the help of a semistructured questionnaire and the guided field-walk method. The informants mentioned a total of 34 plant species that they consumed during Monga. Published literature shows that all the species consumed had ethnomedicinal uses. It is concluded that famine food plants also serve as ethnomedicinal plants.

## 1. Introduction

Human beings need food for survival and to satiate their hunger. Plants have always constituted a major food source for people throughout the world since the advent of humans. During times of natural disasters like inclement weather conditions, populations suffering from severe food shortages become heavily reliant on wild food plants for survival [1]. This has given rise to the concept of famine plants [2]. Rodale and Mcgrath [3] stated that famine plants have been eaten and utilized for centuries. Certain “wild-foods” are enjoyed and therefore collected and consumed every time when ready and these are important “famine-foods” during periods of food shortage [4].

The human population of the western Sahel has been reported to depend on a number of wild plant foods, and this dependency increases during drought conditions [5]. However, scarcity of food or in practicality, famine condition, is also a common occurrence with people who live in poverty and so cannot afford their daily requirements of a normal and conventional diet. Such food scarcity/famine (famine and food scarcity have been considered equivalent in this paper in the sense that both conditions lead to inadequate intake of daily conventional food items) can be observed among the people of Bangladesh, about a third of those who live below the poverty level income, defined as less than US$ 1 per day. Moreover, people of the northern districts of Bangladesh are subjected each year to a seasonal famine known as Monga. Monga usually occurs twice a year; the greater Monga (boro Monga) occurs during the lean season preceding the harvest of paddy in the Bangla months of Ashwin and Kartik (mid-September to mid-November), and the smaller Monga (choto Monga) occurs during the lean season preceding the harvest of paddy in between the Bangla months of Chaitra and Jaistha (mid-March to mid-June). It is to be noted that rice (obtained after dehusking paddy) is the staple cereal of the people of Bangladesh and is the major item consumed by the poorer rural people with lentils and an occasional sidedish of a vegetable. Monga occurs due to a number of factors, lack of adequate water supply during the above months and lack of diversification of jobs (most people being agricultural laborers with little cultivable land of their own). The agricultural laborers, landless farmers, and the marginal farmers suffer from acute food shortage during Monga [6].

Lalmonirhat and Nilphamari districts are two districts in the northern part of Bangladesh, which suffer from Monga. The people in these districts, particularly the rural poor, are the worst sufferers and suffer during Monga from acute food shortages. We have previously shown that a number of nonconventional plant items are consumed by the poor people of the northern districts of Bangladesh during Monga [7]. In fact, such consumption of nonconventional plant items during times of food scarcity has been reported by us for other districts of Bangladesh, like Rangamati and Kurigram [8, 9]; Rangamati district does not suffer from Monga, but food scarcity exists among segments of the mainstream population as well as tribal people. Also notably, Rangamati district is in the southeastern portion of the country. During our survey in Kurigram district on famine food plants, we noted a distinct correlation between nonconventional plants consumed during food scarcity and their folk medicinal usage; in other words, most of the plants consumed had folk medicinal uses [10].

Chronic lack of food causes the people to suffer from malnutrition with consequent wasting away of body and weakening of the body's immune systems [11]. This can cause a number of diseases to occur because of the body's weak defenses against invading pathogens. Lack of proper diet can not only cause shortage of macronutrients like carbohydrates, proteins, and lipids, but also cause lack of vitamins and essential micronutrients with concomitant arising of ailments like anemia, night blindness, beriberi, pellagra, kwashiorkor, and marasmus, to name only a few. Thus nonconventional food items should not only be edible, but also satiate the hunger and meet the body's nutritional needs adequately.

The Australian Aboriginal hunter-gatherers reportedly used to have over 800 plant foods, and that this traditional diet may have been low in carbohydrates but high in fiber, leading to protection of the Aborigines from a genetic pre-disposition to insulin resistance (a physiological condition in which the natural hormone, insulin, becomes less effective in lowering blood sugars) and its consequences like diabetes mellitus, coronary heart disease, and obesity [12]. These conventional food plants and medicine are interrelated as also been shown by other authors. Research in several regions has illustrated that many wild plants that are retained in local food cultures are inseparable from traditional therapeutic systems [13, 14]. Since ancient times, the thinking of “food as medicine” has existed in Chinese medical theories and Chinese food therapy [15, 16]. Etkin and Ross [17, 18] showed from their West African research that many wild plants were used both in therapeutics and for dietary purposes. We further hypothesize that through trial and error, the human population have selected famine food plants items, which not only fulfill hunger satiating and nutritional needs, but also serves a therapeutic purpose. It then follows from our hypothesis that famine food plants, in general, must also have ethnomedicinal uses.

**Figure 1:**
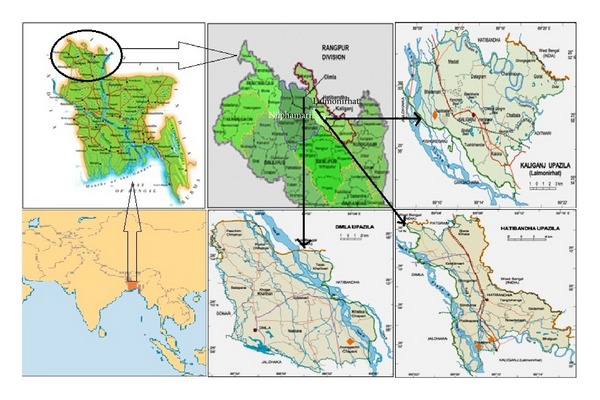
Map of Bangladesh showing the districts, Upazilas, and study sites (villages). Actual study sites are marked on the map as ◆.

The objective of the present survey was to conduct an ethnomedicinal appraisal of famine food plants consumed by poor villagers in four villages (Sailmari, Khurdobichondoi, Paschim Dewwabar, and Schatunama) of two adjoining districts, namely, Lalmonirhat and Nilphamari, which are two of the most Monga-prone districts in Bangladesh, and have substantial segments of the population suffering from food scarcity during Monga. The two districts are bordered on the south by Rangpur district, on the north by West Bengal State of India, on the east by Kurigram district, and on the west by Dinajpur and Panchagarh districts (Figure 1). The area of the four villages where the present survey was carried out approximates 50 square kilometers. An indigenous community, namely the Santals, inhabits portions of the two districts covered. The Santals are considered to be original settlers in this area since prehistoric times; however, the majority of the population (over 98%) of the two districts at present comprises of mainstream Bengali-speaking population.

The villages surveyed lacked any industry; as a consequence, the people are dependent on agriculture. Three of the villages Sailmari, Khurdobichondoi, and Paschim Dewwabar fell under Kaliganj and Hatibandha Upazilas (subdistricts) of Lalmonirhat district, while Schatunuma fell under Dimla Upazila of Nilphamari district (Figure 1). As per National Information Services provided by the Government of Bangladesh [19], the total population of Kaliganj and Hatibanda Upazilas was 216,868 and 239,568, respectively with a literacy rate of 24, and 21.4% (it is to be noted that a person is considered literate in Bangladesh if the person can only sign his or her name without even going to primary school). The total population of Dimla Upazila is 280,076 with an average literacy level of 42.86%. Small farmers (i.e. farmers without land or having less than one-third acre of land per family) constituted over 80% of the population in the villages surveyed; these farmers mostly worked as agricultural laborers in other people's land.

The surveyed villages did not have any forest land. The villages, however, contained fallow land and “char” (river islands on the Teesta River) areas. There was some vegetative cover in the fallow lands and chars; the vegetation mostly consisted of wild herbs, shrubs, and a few trees, which were tropical and subtropical in nature.

## 2. Methods

### 2.1. Study Area and Investigative Methods

The present survey was conducted between October 2010 and August 2012. A preliminary survey was conducted among the villagers of a number of villages in Lalmonirhat and Nilphamari districts, which according to news reports of the country have a substantial number of households, who were affected by Monga. From this preliminary survey, four villages as mentioned above were chosen in the two districts on the basis of the number of households, whose incomes were below the poverty level, and as a consequence, were more affected by Monga. More detailed surveys (comprising of a total of nine visits, each visit lasting four days on an average) were conducted in these four villages among a total of 167 households who mentioned that they consume nonconventional plant items not only during Monga, but also at other times of food scarcity, caused due to their low income levels. All together, 238 adult members (219 females and 19 males) from these households were interviewed. It is to be noted that women, particularly the adult married women members of rural households, are in general responsible for cooking food and collecting nonconventional plant items (during times of food scarcity) and so possess more information on famine foods than the male members of the household. Although collecting nonconventional edible plants from the wild or fallow lands and roadsides is also shared by children along with adult female members of the household, such children were not interviewed in the present survey.

### 2.2. Mode of Interview and Plant Specimen Collection

Informed consent was first obtained from the Head of each household (in most cases being the oldest active male member) to gather information on their monthly income levels, availability of adequate food throughout the year, prevalence of diseases, occupation, literacy, consumption of nonconventional plant food items during times of food scarcity in their households, and the therapeutic uses of the nonconventional plant species. The male Heads of households themselves suggested that information on consumption of nonconventional plants be gathered from the female adult members of each household. Information was collected and recorded with the help of a semistructured questionnaire, open-ended interviews, and the guided field-walk method of Martin [20] and Maundu [21]. In this method, the women informants took the interviewers on guided field walks through areas from where they usually collected their nonconventional edible plants, pointed out the plants, and described the mode of consumption of these plants and the plant parts used for consumption, as well as medicinal values of the plants. All such plant specimens were collected from the spot, pressed, dried [22], and brought back to Dhaka for complete identification by the Bangladesh National Herbarium. Voucher plant specimens were deposited with the Plant Collection Wing of the University of Development Alternative. Nomenclature of plants was compiled from the Plant List database (http://www.theplantlist.org/). Lalmonirhat and Nilphamari are adjoining districts, and it was noticed that the pattern of consumption of nonconventional plants was basically the same for each household in all four villages of the two districts.

### 2.3. Search of Databases for Ethnomedicinal Uses of Plants

Ethnomedicinal uses of the plant reports were collected through searching various databases like PubMed, SCOPUS, and Google Scholar.

## 3. Results and Discussion

### 3.1. Demographic Characteristics

Of the 219 females interviewed, 76 females (34.7%) were in the age group of 21–30 years, 88 females (40.2%) were in the age group of 31–40 years, and 55 females (25.1%) were in the age group of 41–50 years. 100% of the females were married and described their occupation as housewives. The literacy rate among the females was 3.1%. Of the 19 males interviewed, 16 males (84.2%) described their occupation as agricultural laborers, while 3 males (15.8%) described themselves as small farmers with land holding not exceeding 1/3 acres. Literacy rate among the interviewed males was 7.9%. It may be noted that the literacy rate among the surveyed population was observed to be lower than the Upazila average. The informants mentioned that part of this lower literacy rate was due to age, for only recently the Government of Bangladesh has made primary education (up to Grade V) compulsory for both males and females. The other factor mentioned by the informants was that they could not even send children to schools regularly because the children were often engaged in foraging for wild edible plants because of chronic food shortages.

### 3.2. General Dietary Information

According to all informants, their main diet during food availability consisted of rice, which was consumed along with lentil soup (dal), vegetables, and occasionally fish or meat. Since rice contains very low amount of protein, lentils served as the main protein source in the absence of meat or fish items. During times of food scarcity, rice could not be afforded, and so they consumed nonconventional edible plants or plant parts along with lentil soup, if the latter could be afforded. Various types of lentils (pulses) are available in Bangladesh, the most costly being *Lens esculenta* and *Lens culinaris* (masoor dal) and *Vigna radiata* (mung dal). However, the poorer people cannot usually afford these two pulses and consume instead *Lathyrus sativus* (khesari dal).

### 3.3. Plant Habitat

Among the plants whose parts were consumed, with the exception of *Artocarpus heterophyllus*, *Corchorus capsularis*, *Moringa oleifera*, *Musa sapientum*, and *Raphanus sativus*, the rest of the plants were collected from the wild (fallow land, roadsides, open fields, or marshy areas). Aquatic wild edible plants included *Ipomoea aquatica*, *Marsilea minuta*, *Enhydra fluctuans*, *Nelumbo nucifera*, and *Nymphaea pubescens*.

### 3.4. Plants, Plant Parts, and Mode of Consumption during Famines

**Figure 2:**
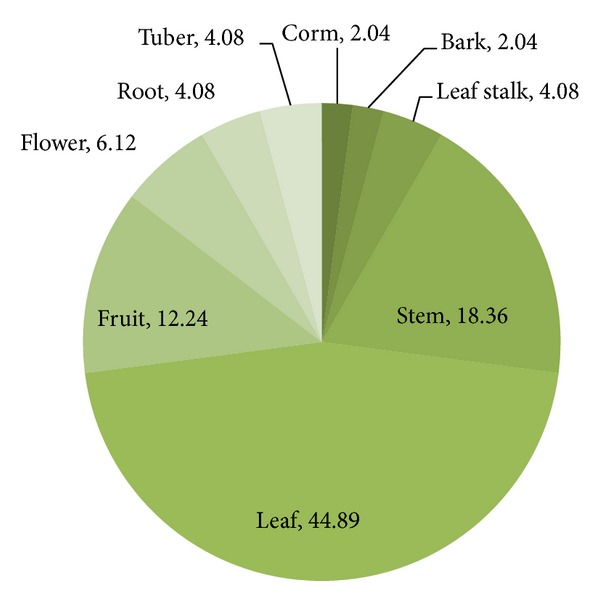
Percentage distribution of various plant parts consumed during times of food scarcity.

The various informants mentioned a total of 34 nonconventional plant species that they consumed during times of food scarcity. The plants were distributed into 26 families. Among these plants species, the parts consumed were leaves, stems, barks, fruits, seeds, flowers, tubers, and corms. The results are shown in Table 1. Leaves formed the major plant part consumed and constituted 44.9% of the total. Leaves were followed by stems at 18.3% and fruits at 12.2%. The results are shown in Figure 2. In other parts of the world like Niger in Africa, leaves have been reported to be primarily consumed during famines and have been shown to be excellent sources of proteins and micronutrients, particularly of plants like *Amaranthus viridis* and *M. oleifera* [23]. Notably, the leaves of these two plants were also found to be consumed by the people of the present survey areas during times of food scarcity.

Fruits were usually eaten directly in the raw state, tubers and corms in the mashed state following boiling in water, and leaves and stems taken following frying or cooking in the form of vegetables. Since the households were too poor to afford spices, essentially a little oil or a small amount of table salt was added for cooking and making the dish more palatable. The three exceptions to this generalized mode of consumption were *Centella asiatica*, *M. minuta*, and *Oxalis corniculata*. In all these three cases, juice obtained from squeezed leaves was added to lentil soup, which was then consumed. The reason for this unusual mode of consumption was attributed to age-old practices of the community. Among these plants, not all plant parts consumed were fully nonconventional. For instance, during regular times of food availability, villagers would consume leaves and stems of *Amaranthus tricolor*, seeds of *A. heterophyllus*, leaves of *C. asiatica*, leaves, stems, and tubers of *Colocasia esculenta*, leaves of *C. capsularis*, leaves of *I. aquatica*, fruits of *Musa paradisiaca*, leaf stalks of *N. pubescens*, and leaves of *R. sativus*, but only occasionally. *M. sapientum* and *Musa paradisiaca* fruits were consumed during regular times, but during times of food scarcity, other parts of the plant along with fruits were consumed.

### 3.5. Food Uses of Famine Food Plants of Surveyed Areas in Other Parts of the World

It is of interest that the plants consumed during times of food scarcity in the surveyed areas are also used as normal or famine foods in other regions of the world, although the same plant part may not be consumed. Food uses of some of the plants are shown in Table 2. For instance, leaves and seeds of *Abroma augusta* are considered edible in Papua New Guinea and Sikkim, India, respectively [24, 25]. The surveyed population consumed the barks and roots of the plant. Leaves of *Alternanthera sessilis* are also considered edible in Papua New Guinea [24]; the surveyed population consumed leaves and stems. Leaves of *Amaranthus spinosus* and *Amaranthus viridis* are eaten as leafy vegetables in Assam, India [26]; the local people consumed both leaves and stems. The flowers of *Bombax ceiba* are considered edible in Arunachal Pradesh, India [27]; the local people consumed the roots of the plant.

### 3.6. Local Ethnomedicinal Uses of Plants Consumed during Famines

**Figure 3:**
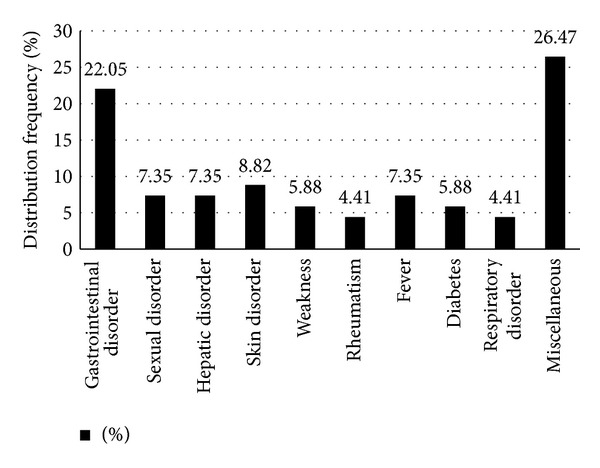
Frequency distribution (%) of famine food plants used for treatment of various medical disorders. Besides the disorders shown in the figure, other disorders for which famine food plants were used have been classified in general as Miscellaneous.

With the exception of five plants, the rest 29 plants (85.29% of total) were reported by the informants to have medicinal uses. The frequency of plant use in different categories of disorders is shown in Figure 3. Gastrointestinal disorders had the maximum frequency of ethnomedicinal use (22.05%), followed by skin disorders (8.82%). Other major disorders against which plants were reported to have ethnomedicinal uses included sexual disorders, hepatic disorders, and fever (7.35% each). The rural population and particularly the surveyed rural poor households were found to live under unhygienic conditions and with poor sanitation and drinking water quality. These factors along with possible fall of immunity due to malnutrition [11] can lead to various diseases, and gastrointestinal disorders and skin diseases would constitute the major disease forms. Other studies have also indicated the prevalence of gastrointestinal disorders among the Bangladeshi rural population [28, 29].

### 3.7. Ethnomedicinal Uses of the Famine Food Plants in Other Regions of the World Including Bangladesh

To validate our hypothesis that, through trial and error, the human population has selected famine food plants items, which not only fulfill hunger satiating and nutritional needs, but also serves a therapeutic purpose, it was of interest to examine published reports on ethnomedicinal uses of the famine food plants of the survey areas in other parts of the world, including Bangladesh. The results are presented in Table 3. It is to be noted that only a selection of available reported ethnomedicinal uses of the plants are presented in Table 3. Not surprisingly, the ethnomedicinal uses of the local famine food plants were much greater when other regions of the world were taken into account. However, some local medicinal uses were in common with uses in other regions (i.e., treatment of menstrual problems with *A. augusta* or use of *C. asiatica* for treatment of gastrointestinal disorders). 

Taken together, the available ethnomedicinal reports on the nonconventional plants consumed by the villagers surveyed, strongly validates our hypothesis that famine food plants are also ethnomedicinal plants. That the exact ethnomedicinal value be actually known is possibly not necessary; just the mere observation that consumption of these plants satiate hunger, meet nutritional needs to a lesser or greater extent, and somehow prevents diseases from occurring can be valid reasons for selection of particular nonconventional plants and not others. For instance, the informants did not mention any medicinal uses for the plants, namely, *Caryota urens*, *Ehretia acuminata*, *Malva verticillata*, *N. pubescens*, and *R. sativus* (Table 1); however, all four plants have reported ethnomedicinal uses in other parts of the world (Table 3). The reasons for discarding other wild or nonconventional plants can be due to a variety of reasons ranging from toxicity and lesser palatability to lesser fulfillment of nutritional needs, with all these factors being easily manifested.

The question then arises as to why did not the villagers surveyed in the present study consume these nonconventional plant items on a regular basis? One answer provided by the villagers themselves was that they did not find these nonconventional plant items as palatable (in their words tasty) as their regular diet of rice and lentils. A further answer could be that they were unaware of all the health benefits that these nonconventional plants offered (as also suggested from a comparison of local medicinal uses of the plants versus ethnomedicinal uses in other parts of the world), and so they stuck to their millennia old dietary habits. Another possible reason could be that they once were aware of the ethnomedicinal benefits of the plants consumed but have lost some of that knowledge over time. In fact, “optimum foraging strategy” theory [30] implies that all animals forage in such a way as to maximize their net energy intake per unit time. We extend this hypothesis to include that humans forage or rather use famine food plants in such a manner which besides maximizing their net energy intake per unit time also provides them with health benefits in the form of preventing or curing diseases. This also makes sense; during malnutrition arising out of food scarcity, humans may have reduced strength and weakened body defenses; as such, they would include food, which would offer both nutritional as well as therapeutic benefits. It is interesting to note that another study in Northeast Thailand also found that half of the weedy vegetables consumed by the people are also regarded as sources of medicine [31]. That various wild plants can serve as both food and medicine has been reported from various regions of the world including Palestine and China [32, 33].

The major finding of this study is that, since famine food plants have the real possibility of multiple ethnomedicinal uses, such plants throughout the world merit further scientific studies to fully explore their medicinal potentials. Moreover, since famine food plants are mostly wild but edible and can grow under inclement weather conditions without any particular care [1], they can potentially be sources of both future foods and medicine.

## 4. Conclusion

Famine food plants have generally been mentioned as unconventional dietary items and consist of wild edible plants. It was our hypothesis that such plants also serve therapeutic purposes and can be considered ethnomedicinal plants. Through local surveys among famine-affected population of two districts of Bangladesh on the unconventional plants they consume during famine periods, along with local and other reported ethnomedicinal uses on these plants, we have validated our hypothesis.

## Figures and Tables

**Table 1:** Nonconventional (famine) food plants of four villages in Lalmonirhat and Nilphamari districts, Bangladesh.

Serial number	Species	Family	Local name (English name)	Part(s) used	Mode of consumption	Local medicinal use(s)
1	*Abroma augusta* (L.) L.f.	Malvaceae	Ulot kombol (Devil's cotton)	Bark, root	Juice obtained from crushed bark is taken with a little salt. Smashed roots are cooked.	Roots used against menstrual problems, leucorrhea, stomach pain, and sexual weakness. Bark used against jaundice.

2	*Alternanthera sessilis* (L.) R. Br. ex DC.	Amaranthaceae	Shanti shak (Sessile joyweed)	Leaf, stem	Fried with little oil and water.	Leaves used against scabies and eczema.

3	*Amaranthus spinosus* L.	Amaranthaceae	Kanta khuria (Spiny Amaranth)	Leaf, stem	Fried with little oil and water.	Leaves and stems used against boils, stomach pain, and weakness.

4	*Amaranthus tricolor* L.	Amaranthaceae	Chorie danga shak (Joseph's coat Amaranth)	Leaf, stem	Fried with little oil and water.	Leaves and stems used against skin diseases and dysentery.

5	*Amaranthus viridis* L.	Amaranthaceae	Khai khuria (Green Amaranth)	Leaf, stem	Fried with little oil and water.	Leaves and stems used against boils, constipation, and severe malnutrition.

6	*Artocarpus heterophyllus* Lam.	Moraceae	Kanthal (Jackfruit)	Seed	Roasted seeds are eaten directly or in the mashed form.	Seeds used against constipation.

7	*Bombax ceiba* L.	Bombacaceae	Shimul (Silk cotton tree)	Root	Cut into small pieces and boiled with a little salt.	Roots used against sexual weakness.

8	*Caryota urens* L.	Arecaceae	Cha guwa (Solitary fishtail palm)	Fruit	Fruits are eaten raw.	No local medicinal uses reported.

9	*Centella asiatica* (L.) Urb.	Apiaceae	Khudi manimuni (Indian pennywort)	Leaf	Juice obtained from crushed leaves is added to lentil soup.	Leaves used against indigestion, diarrhea, and dysentery.

10	*Chenopodium album* L.	Chenopodiaceae	Bothua (Lamb's quarters)	Leaf, stem	Fried with little oil and water.	Leaves and stems used against liver diseases, helminthiasis, hemorrhoids, constipation, and bloating.

11	*Colocasia esculenta* (L.) Schott.	Araceae	Kochu (Elephant's ear)	Leaf, stem, tuber	Leaves and stems are fried with little oil and water. Tubers are boiled in water containing salt and consumed in the mashed form.	Leaves and stems used against indigestion. Tubers used against tuberculosis, rheumatism, and paralysis.

12	*Corchorus capsularis* L.	Tiliaceae	Paat shak (Jute)	Leaf from young plants	Cut into small pieces and cooked with little salt and water.	Leaves used against stomach pain, liver disorders, and loss of appetite.

13	*Dioscorea esculenta* (Lour.) Burkill	Dioscoreaceae	Boro alu (Lesser yam)	Leaf, tuber	Boiled in water and then taken in the mashed form after mixing with oil and hot peppers.	Tubers used against malnutrition.

14	*Diplazium esculentum* (Retz.) Sw.	Dryopteridaceae	Dhakia shak (Vegetable fern)	Leaf	Cut into small pieces and cooked.	Leaves used against fever.

15	*Ehretia acuminata* R. Br.	Boraginaceae	Kath guwa (Kodo weed)	Fruit	Fruits are eaten raw.	No local medicinal uses reported.

16	*Enhydra fluctuans* Lour.	Asteraceae	Hanchi shak (Water cress)	Leaf	Fried with salt and then cooked.	Leaves used against diabetes, low semen density, and weakness.

17	*Ficus hispida* L.	Moraceae	Khoksha (Hairy fig)	Fruit	Fruits are eaten raw.	Fruits used against diabetes and hypertension.

18	*Glinus oppositifolius* (L.) Aug. DC.	Molluginaceae	Teeta shak (Bitter leaf)	Leaf	Leaves are boiled in water followed by discarding the water and cooking the boiled leaves as vegetable.	Leaves used against indigestion, fever, and burning sensations in hands or feet.

19	*Ipomoea aquatica* Forssk.	Convolvulaceae	Kolmi shak (Water spinach)	Leaf	Cut into small pieces and cooked with salt and water.	Leaves used against chicken pox and rheumatism, and to increase lactation in nursing mothers.

20	*Ipomoea batatas* (L.) Poir.	Convolvulaceae	Misti aloo (Sweet potato)	Leaf	Cut into small pieces and cooked with salt and water.	Leaves used against diarrhea and debility.

21	*Leucas aspera* (Willd.) Link	Lamiaceae	Kanshika (White dead nettle)	Leaf from young plants	Cut into small pieces and cooked with a little salt.	Leaves used against body pain, coughs, and mucus.

22	*Malva verticillata* L.	Malvaceae	Napa shak (Chinese mallow)	Leaf	Cooked with little water and salt.	No local medicinal uses reported.

23	*Marsilea minuta* L.	Marsileaceae	Dhel manimuni (Dwarf water clover)	Leaf	Leaves are squeezed to obtain juice, which is consumed with lentil soup.	Leaves used against edema, sexual weakness, mucus and fever.

24	*Moringa oleifera* Lam.	Moringaceae	Saazna (Drumstick tree)	Leaf, immature fruit	Leaves are cooked with a little soda and consumed. Fruits are cooked as vegetables.	Leaves and fruits used against fever, boils, cold, and joint pain.

25	*Musa x paradisiaca* L.	Musaceae	Anajee kola (Plantain)	Flower, fruit, pseudostem	Immature flowers are taken in the mashed form with a little salt after boiling. Unripe fruits are boiled and taken in the mashed form or cooked as vegetable. Pseudostems from young plants are cooked as vegetable.	Fruits used against anemia, hematemesis, and dysentery. Flowers and pseudostems used against chronic dysentery.

26	*Musa x sapientum *L.	Musaceae	Aeeta kola (Banana)	Flower, fruit	Immature flowers are boiled and taken in the mashed form with a little salt. Ripe fruits are taken raw or smashed and kept in water for 10–12 hours followed by drinking the mixture.	Fruits against stomach pain, diarrhea, and skin eruptions. Flowers used against diabetes.

27	*Nelumbo nucifera* Gaertn.	Nelumbonaceae	Padma, Dhepra (Sacred lotus)	Leaf stalk	Leaf stalks are fried with a little salt.	Leaves and leaf stalks used against weakness.

28	*Nymphaea pubescens* Willd.	Nymphaeaceae	Shapla, Shaluk (Hairy water lily)	Leaf stalk, corm	Leaf stalks are fried with a little salt. Corms are roasted and taken with a little salt.	No local medicinal uses reported.

29	*Oxalis corniculata* L.	Oxalidaceae	Amrul (Creeping woodsorrel)	Leaf	Juice obtained from crushed leaves is taken with lentil soup.	Leaves used against dysentery and as antidote to poison.

30	*Raphanus sativus* L.	Cruciferae	Mula (radish)	Leaf	Cut into small pieces and cooked in water with some salt and a little oil.	No local medicinal uses reported.

31	*Saccharum spontaneum* L.	Poaceae	Keshur, Kashia danda (Wild sugar cane)	Stem	Stems are chewed and the ensuing juice is taken orally.	Stem juice used against jaundice and sexual weakness.

32	*Scoparia dulcis* L.	Scrophulariaceae	Misti pata (Sweet broomweed)	Leaf	Leaves are cooked with a little salt.	Leaves used against fever, dysentery, blood dysentery, and gastric ulcer.

33	*Sesbania grandiflora* (L.) Pers.	Fabaceae	Bokful (August flower)	Flower	Fried.	Flowers used against biliary disorders and diabetes.

34	*Spilanthes paniculata* Wall. ex DC.	Asteraceae	Oshun shak, Roshun shak (Para cress)	Leaf, stem	Cut into small pieces and cooked.	Leaves and stems used against rheumatism.

**Table 2:** Reported food uses of the famine food plants.

Species	Use as food plant
*Abroma augusta* L.	Leaves are considered edible in Papua New Guinea [24] and seeds in Sikkim, India [25].
*Alternanthera sessilis* (L.) R. Br. ex DC.	Leaves are considered as wild edibles in Papua New Guinea [24].
*Amaranthus spinosus* L.	Consumed as leafy vegetable in Assam (India) [26].
*Amaranthus tricolor* L.	Considered an edible vegetable in North India [34].
*Amaranthus viridis* L.	Consumed as leafy vegetable in Assam (India) [26].
*Artocarpus heterophyllus* Lam.	Fruits and seeds consumed in Malaysia [35].
*Bombax ceiba* L.	Flowers eaten as vegetable in Arunachal Pradesh of India [27].
*Caryota urens* L.	Pith used as famine food in South India [36].
*Centella asiatica* (L.) Urb.	Considered a leafy vegetable in Assam (India) [26].
*Chenopodium album* L.	Considered a leafy vegetable in Assam (India) [26].
*Colocasia esculenta* (L.) Schott.	Considered a leafy vegetable in Assam (India) [26].
*Corchorus capsularis* L.	Leaves are eaten in the cooked form in some Asian countries [37].
*Dioscorea esculenta* (Lour.) Burkill	Tubers are reported as wild edible in the islands of Remote Oceania [38].
*Diplazium esculentum* (Retz.) Sw.	Leaves consumed in Yunnan, China [39].
*Ehretia acuminata* R. Br.	Fruits are eaten raw by aboriginals in Australia [40].
*Enhydra fluctuans* Lour.	Leaves and stems consumed as leafy vegetable by ethnic communities in Tripura, India [41].
*Ficus hispida* L.	Fruits are eaten raw in Arunachal Pradesh of India [27].
*Glinus oppositifolius* (L.) A. DC.	Young leaves and stems consumed as vegetable in West Bengal, India [42].
*Ipomoea aquatica* Forssk.	Leaves and stems are cooked and consumed in Malaysia [35].
*Ipomoea batatas* (L.) Lam.	Leaves and stems are cooked and consumed in Malaysia [35].
*Leucas aspera* (Willd.) Link	Young leaves consumed during famine in Kurigram district, Bangladesh [10].
*Malva verticillata* L.	Young leaves consumed as soup in Korea [43].
*Marsilea minuta* L.	Leaves and stems consumed as vegetable in Jharkand, India [44].
*Moringa oleifera* Lam.	Leaves, fruits, flowers consumed in the cooked form in many countries of South Asia and Africa [45].
*Musa paradisiaca* L.	Fruits consumed in the unripe state in tropical countries [46].
*Musa sapientum* L.	Ripe fruits consumed throughout the world [47].
*Nelumbo nucifera* Gaertn.	Consumed as vegetable in various parts of India [48].
*Nymphaea pubescens *Willd.	Roasted endosperm consumed by rural communities in Assam, India [49].
*Oxalis corniculata* L.	Consumed by tribal communities of Central India during times of food scarcity [50].
*Raphanus sativus* L.	Dietary vegetable in Asian countries, particularly China, Japan, and Korea [51].
*Saccharum spontaneum* L.	Stems used to mitigate thirst or hunger by tribes in Parambikulam Wildlife Sanctuary, Kerala, India [52].
*Scoparia dulcis* L.	Consumed as vegetable in northeastern Thailand [53].
*Sesbania grandiflora* (L.) Pers.	Flowers and buds consumed as vegetable in India [54].
*Spilanthes paniculata* Wall. ex DC.	Special food item prepared from the plant during religious festivals by the Mising community of Assam, India [55].

**Table 3:** Reported ethnomedicinal uses of the non-conventional food plants shown in Table 1.

Serial number	Species	Reported ethnomedicinal uses	Reported pharmacological activities
1	*Abroma augusta* L.	Root juice taken orally by tribal and rural people in West Rarrh region of West Bengal, India, for blood dysentery, diarrhea, and night wetting [56]; powder of bark and roots consumed thrice a day with boiled water by the Khamti tribe of Arunachal Pradesh, India, for urinary problems [57]; flowers taken orally by the Tonchongya tribe of Bandarban district, Bangladesh, for mental sickness [58]; stem juice to be taken orally, advised by folk medicinal practitioners in Begumganj, Noakhali district, Bangladesh, for irregular menstruation, painful menstruation, and burning sensations in the uterus [59]; root juice taken orally as uterine tonic by ethnic communities of Tinsukia district of Assam, India [60]; leaf juice orally taken, advised for diabetes by folk medicinal practitioners of Vasu Bihar village, Bogra district, Bangladesh [61]; crushed stems taken orally in Vitbilia village of Pabna district, Bangladesh, for treatment of debility and infertility in women due to uterine problems [62]; leaf juice taken orally for diabetes and root juice for sexual disorder by the Garo tribal community of Netrakona district, Bangladesh [63]; leaf juice taken orally for heatstroke in Brahmanbaria district, Bangladesh [64].	Antidiabetic, antioxidant, anti-inflammatory, wound healing, hypolipidemic, antifungal, antibacterial, insecticidal, uterine disorders [65].

2	*Alternanthera sessilis* (L.) R. Br. ex DC.	Whole plant used by ethnic groups in Sialkot district, Pakistan, for headache, dizziness, snake bite, and vomiting of blood [66]; plant along with leaves of *Hibiscus rosa sinensis* tied with a piece of cloth by ethnic groups of Disoi valley forest area of the Jorhat district of Assam, India, for bone fracture and wounds [67]; dried leaves used for treatment of jaundice by Palliyar tribals in Sirumalai Hills, Western Ghats, Tamil Nadu, India [68]; root, stem, and leaf decoction taken orally by the tribals of Bargarh district, Orissa, India, for blood dysentery [69]; whole plant used for treatment of wounds by different tribal communities of Uttara Kannada district, Karnataka, India [70]; leaves and whole plants local tribal people of Kaptipada Forest Range, Orissa, India, for treatment of fevers, ophthalmia, gonorrhea, and pruritis [71]; whole plant used by the local people of Amarkantak region, Madhya Pradesh, India, for burning sensation, diarrhea, skin disease, dyspepsia, hemorrhoids, liver and spleen diseases, and fever [72]; plant juice orally used against chronic dysentery in Jajpur district, Odisha, India [73]; leaves used in Noakhali district, Bangladesh, for treatment of gonorrhea, low semen count, and leucorrhea [59]; leaves used by local Irula tribals of Kalavai village, Vellore district, Tamil Nadu, India, for treatment of headache, hepatitis, and asthma [74]; leaves used by people of Alagar Hills, Eastern Ghats, Tamil Nadu, India, for treatment of eyesight [75]; leaf juice used by the Nath people of Assam, India, to increase lactation in nursing mothers and for treatment of hair and stomach trouble [76].	Anti-inflammatory [77] hematinic [78], wound healing [79], antidiabetic [80].

3	*Amaranthus spinosus* L.	Leaves are boiled in cow milk and orally taken in Kikuku village, Muleba district, Tanzania, for treatment of peptic ulcers [81]; fresh root infusion along with salt orally taken for throat infections by the Tripuri tribes of Tripura State, India [82]; root paste applied topically for eczema or abscesses in Jajpur district, Odisha, India [73]; root paste topically used by Hooralis tribe in Sathyamangalam forests of Western Ghats, Tamil Nadu, India, for wounds and blisters [83]; decoction of whole plant orally taken for treatment of HIV/AIDS at Tokombere (far north Cameroon) [84]; ash of whole plant taken orally for treatment of kidney stones; fresh leaves are cooked along with chicory plant and fenugreek and taken orally for low blood pressure and black cataract of eye by local communities in arid regions of Pakistan [85]; leaf juice along with leaf juice of *Mangifera indica* and whole plant juice of *Sida rhombifolia* used by the Tripura tribal community of Comilla district, Bangladesh, for treatment of jaundice [86]; root, bark, and stem orally taken in Kurigram district, Bangladesh, for stoppage of urination and defecation [87]; upper parts of the plant used as a febrifuge by the tribes of Lalganj block of district Mirzapur, Uttar Pradesh, India [88]; used for menorrhagia, gonorrhea (roots), and snake bite (roots) and to increase milk flow in cows (stems) by the Nath people of Assam, India [76].	Antiprotozoal, anti-inflammatory, antioxidant, antimalarial, analgesic, immunomodulatory, hepatoprotective, antifertility, antidiabetic, antihyperlipidemic [89].

4	*Amaranthus tricolor* L.	Whole plant used in Kurigram district, Bangladesh, for treatment of anemia and “meho” (diabetes) [87]; whole plant used in Ivanur Panchayat in Cuddalore district, Tamil Nadu, India, to improve eye power [90]; curry prepared from green leaves taken orally to stop diarrhea; seeds taken orally for general gastric problems; seeds fried in butter taken orally to lessen pregnancy pains by the Lepcha tribe of Dzongu valley, bordering Khangchendzonga Biosphere Reserve, in North Sikkim, India [91]; whole plant used by the local people of Mansoora, Malegaon, India, as astringent and for treatment of menorrhagia, diarrhea, and dysentery [92]; leaf paste applied topically by ethnic communities of Tinsukia district, Assam, India, for cuts and wounds [60]; leaf paste used to cure wounds in Darikal Gaon of Tezpur in Assam, India [93]; leaves cooked and eaten as vegetable as treatment for anemia in Semiliguda block of Koratpur district, Odisha, India [94].	Hepatoprotective, nutritive, blood tonic [95].

5	*Amaranthus viridis* L.	Whole plants and stems used for treatment of bronchitis, piles, leucorrhea, breast abscess, menorrhagia by local tribal people of Kaptipada Forest Range, Orissa, India [71]; used for boils (roots) and malnutrition in pregnant mothers (leaves and stems are cooked and eaten) in Kurigram district, Bangladesh [87]; root decoction used by tribals of Samahni Valley, Pakistan, to control menstrual problems and backbone ache during pregnancy [96]; plant used against cough, inflammation, high blood pressure, and as urinative by people in arid regions of Pakistan [85]; leaves used against stomach colic and as laxative by tribals of Darjeeling Hills, India [97]; leaves taken orally for dysentery, as a diuretic, and to alleviate internal fever in Nizamabad district, Andhra Pradesh, India [98]; used against snake bite (stem) and scorpion sting (leaf) by the Nath people of Assam, India [76]; whole plant used in Bhopal district, India, for treatment of stone diseases [99]; tender shoots taken as vegetable to improve eyesight by the ethnic communities of Tinsukia district, Assam, India [60]; leaf juice taken orally for chronic dysentery in villages of Natore and Rajshahi districts, Bangladesh [100].	Antinociceptive, antipyretic, blood tonic [95].

6	*Artocarpus heterophyllus* Lam.	Peduncle juice taken orally thrice daily for snake bites in West Rarrh region of West Bengal, India [56]; latex applied topically as treatment of skin disease, wound, and scorpion sting in Jorhat district, Assam, India [67]; leaves used for skin diseases, ulcer, asthma, and diarrhea in Tamil Nadu, India [101]; ash of rind spine applied topically on throat or tongue for treatment of ulcers in Nasik district, Maharashtra, India [102]; used against bloating (unripe fruit), constipation (ripe fruits), edema, ulcers (leaf ash), skin diseases (topical application of young leaf and roots), asthma, and diarrhea in Noakhali district (oral administration of young leaves and roots), Bangladesh [59]; used against gastrointestinal disorders in Iloilo, the Philippines (plant parts not mentioned) [103].	Antioxidant, anti-inflammatory, antibacterial, anticariogenic, antifungal, antineoplastic, antidiabetic, wound healing [104].

7	*Bombax ceiba* L.	Stem bark used for treatment of herpes infection in Coastal Karnataka, India [105]; skin diseases, female diseases, and snake bite in Manipur, India (plant part used not mentioned) [106]; leaves are soaked in water and the decoction used for taking a bath for treatment of body pain by the Orang Asli in Kampung Bawong, Perak, West Malaysia [107]; decoction of root used by the people of Kadhi areas of Khushab, Punjab, Pakistan, to kill abdominal worms [108]; leaf paste applied topically for treatment of snake bite by the Mullu kuruma tribe of Wayanad district, Kerala, India [109]; seed used by tribals of Chitteri Hills, India, to treat diabetes [110]; flower paste applied topically by the Chakma communities of Chittagong Hill Tracts, Bangladesh, for treatment of boils [111]; roots of the plant taken orally with seeds of *Hyptis suaveolens* by the Marakh sect of the Garo tribe in Mymensingh district, Bangladesh, against gonorrhea [112]; used in Samba district of Jammu and Kashmir against diarrhea, dysentery, menorrhagia, stomach complaints, diabetes, menstrual disorders, and for conception, and as an aphrodisiac (plant part used not mentioned) [113]; root used against diabetes by the tribes of Pedabayalu Mandalam, Visakhapatnam district, Andhra Pradesh, India [114]; used against urinary problems (fruits) and diarrhea (stem bark juice) by the Gond tribe of Adilabad district, Andhra Pradesh, India [115].	Aphrodisiac, anti-inflammatory, hepatoprotective, anticancer, anti-HIV, anti-*Helicobacter pylori*, antiangiogenic, analgesic, antioxidant, hypotensive, hypoglycemic, antimicrobial [116].

8	*Caryota urens* L.	Decoction of root used as a galactagogue by nursing mothers in Tinsukia district, Assam, India [60]; inflorescence juice and nut exudates used for asthma, as a mild laxative, and as a coolant by the aboriginals of Kalrayan and Shervarayan Hills, Easten Ghats, Tamil Nadu, India [117]; toddy prepared from plant sap used by the Gonds of Adilabad district, Andhra Pradesh, India, to heal urinary problems [115]; ash prepared by burning old leaves is orally taken with honey for treatment of tympanitis (inflammation of middle ear) by the tribals of Similipal Bioreserve, Orissa, India [118].	Antioxidant [119].

9	*Centella asiatica* (L.) Urb.	Leaves either eaten as paste or cooked and eaten as vegetable against hepatic diseases like jaundice, cirrhosis, and liver injury by the Halam tribe of Tripura State, India [120]; whole plant juice orally taken for syphilis and ulcer by the Chakma tribal communities of Chittagong Hill Tracts region, Bangladesh [111]; used against leucorrhea and eczema by the Malasars tribal healers of Velliangiri Hills, India (plant part used not mentioned) [121]; whole plant cooked and eaten for treatment of stomach disorder by the Boro tribe of Manas National Park, Assam, India [122]; whole plant used for treatment of herpes in Coastal Karnataka, India [105]; decoction of stems and leaves taken orally for cough relief and crushed leaves and stems applied to burns by the Kalanguya tribe in Tinoc, Ifugao, Luzon, Phillipines [123]; leaves are grounded with fresh turmeric and applied against skin diseases by the Kurichyas tribe in Kannur district, Kerala, India [124]; whole plant used against fever and sunstroke by the Manavalakuruchi people of Kanyakumari district, Tamil Nadu, India [125]; paste of whole plant used against carbuncle; crushed leaves are mixed with resin from *Artocarpus heterophyllus* and taken orally with fire-roasted *Channa punctatus* fish for treatment of piles by the Tai-Khamyang tribe of Assam, India [126]; leaf juice taken orally for blood purification, blood clots, and appendicitis by the Kani tribals in Pechiparai forests of Southern western Ghats, Tamil Nadu, India [127]; leaf juice used by the tribal communities of Chitrakoot, Madhya Pradesh, India, against rickets in children [128]; used against syphilis, mental disorders, and skin diseases by the Baiga tribals in Amarkantak Meikal forest of Madhya Pradesh, India (plant part not mentioned) [129]; leaves used against rheumatism and dysentery by tribals and local inhabitants of Rajouri-Poonch of Jammu and Kashmir State, India [130]; plant juice orally taken by the Marakh sect of the Garo tribe in Mymensingh district, Bangladesh, against excessive bleeding during menstruation [112]; raw roots and leaves are taken orally with routine food by the Aka tribe of West Kameng district, Arunachal Pradesh, India, to improve appetite during jaundice [131]; dried and powdered whole plants orally taken against azoospermia and streptospermia in Bansoa, West Cameroon [132]; decoction of whole plant applied topically along with coconut oil against wounds by the Malayali tribes of Pachamalai Hills, Tamil Nadu, India [133].	Antiulcer, wound healing, antitumor, memory enhancing, neuroprotective, cardioprotective, hepatoprotective, antioxidant, immunomodulatory, radioprotective, antidepressant, antipsoriatic, antitubercular, antileprotic, antifilarial, antiviral, antiprotozoal, sedative, antispasmodic, anti-inflammatory [134].

10	*Chenopodium album* L.	Whole plant used as laxative to cure constipation by the inhabitants of northern part of Nara desert, Pakistan [135]; decoction of leaves and stems cooked as vegetable and taken orally against tuberculosis, jaundice, fevers, glottis pain, flu, phlegm, dropsy, inflammation, kidney, and gall bladder stones, and as diuretic, blood purifier, and caloric among local communities in arid regions of Pakistan [85]; whole plant used against jaundice by inhabitants of Jalalpur Jattan, Punjab, Pakistan [136]; whole plant used against anemia and constipation by tribals and local inhabitants of Rajouri-Poonch of Jammu and Kashmir State, India [130]; decoction of whole plant used as diuretic and for women's sterility in traditional medicine of east Anatolia, Turkey [137]; whole plant used against rheumatism/arthritis in Betul district, Madhya Pradesh, India [138]; whole plant used against jaundice and liver diseases in Mandi Bahaudin district, Pakistan [139]; seeds used as stimulant, diuretic, carminative, and antispasmodic by tribes of Hamirpur valley, Himachal Pradesh, India [140]; used in Bhopal district, India, for treatment of stone diseases (plant part used not mentioned) [99]; cooked leaves used in urinary troubles, and colic pain; leaf extract used in piles, coughs, and worms; stem used in kidney stone, hepatic disorder, jaundice, and as a galactagogue; whole plant used as laxative; root powder used in spermatorrhea in Sialkot district, Pakistan [141]; tender shoots used against constipation and coughs by ethnic communities of Tinsukia district, Assam, India [60]; whole plant except root for prevention of hemorrhoids (piles) in Chuadanga district, Bangladesh [142].	Antipruritic, antinociceptive [143].

11	*Colocasia esculenta* (L.) Schott.	Whole plant along with bulb of *Allium sativum* and bark of *Cinnamomum verum* is cooked with turmeric and ginger followed by separation of the liquid portion, which is then orally taken against rheumatism and debility in Dinajpur and Thakurgaon districts, Bangladesh [144]; tubers are fried in mustard oil and taken as vegetable against rheumatic pain and paralysis in three villages of Kurigram district, Bangladesh [87]; leaves used by tribals of Chitteri Hills, India, to cure piles [110]; leaves used against jaundice by the Tai-Khamyangs of Assam, India [126]; leaves fried in castor oil used for relieving joint pain by the Gond tribe of Bhandara district, Maharashtra, India [145]; raw leaves are orally taken by the Paliyan and Pulayan tribes of lower Palni Hills of Tamil Nadu, India, against stone formation in the urinary tract and for frequent urination [146]; rhizome paste applied in cuts, burns, and scorpion stings by ethnic groups of Disoi valley forest area of the Jorhat district of Assam, India [67]; paste prepared from tuber is used topically against swellings and cooked rhizome is eaten for helmintic infestations by the Kattunayakas tribes of Mudumalai Wildlife Sanctuary, Nilgiris district, Tamil Nadu, India [147]; whole plant used against severe jaundice, constipation, and as a digestive aid in Shitol Para village of Jhalokati district, Bangladesh [148].	Hypoglycemic, antifungal anticancer, hypolipidemic, anti-inflammatory, nervine tonic [149].

12	*Corchorus capsularis* L.	Fresh leaf decoction administered orally against stomach ache in children in North Bengal, India [150]; leaf juice orally taken to cure dysentery by tribals of Bargarh district, India [69]; seeds and leaves used as stomachic by Bhil tribe of Bibdod, Madhya Pradesh, India [151]; seeds used as stomachic by the tribes of Pedabayalu Mandalam, Visakhapatnam district, Andhra Pradesh, India [114].	Cardioprotective, antiinflammatory, antinociceptive [152].

13	*Dioscorea esculenta* (Lour.) Burkill	Tuber is applied as poultice on swellings by ethnic communities of Tinsukia district of Assam, India [60].	Antioxidant [153].

14	*Diplazium esculentum* (Retz.) Sw.	Boiled young fronds are taken with boiled rice as laxative by the Adi tribes of Dehang-Debang Biosphere Reserve in Arunachal Pradesh, India [154]; roots are boiled in water till the volume is 1/4th of the original volume; 3 mL of the decoction is taken with 2 mL honey on an empty stomach for 15 days against spermatorrhea by tribal communities in Similipal Biosphere Reserve, Orissa, India [155]; decoction of rhizome is orally taken against haemoptysis and coughs by tribal communities of Poba Reserved Forest, Assam, India [156]; juice obtained from a handful of leaves is orally taken to get relief from cold and coughs by inhabitants of Kolli Hills, Eastern Ghats, Tamil Nadu, India [157]; macerated roots are used against skin disorders in Rajbari district, Bangladesh [158]; macerated bark of roots orally taken for detoxification of medicine overdosage by the Tonchongya tribe in Bandarban district, Bangladesh [159]; leaves used to treat headache by indigenous people of Manokwari, West Papua [160].	Antioxidant, central nervous system stimulant [161].

15	*Ehretia acuminata* R. Br.	Extract of leaves mixed with water and taken orally once daily for 2-3 days against dysentery by Chorei tribes of southern Assam, India [162].	None reported.

16	*Enhydra fluctuans* Lour.	Leaf and twig extract taken with equal amount of Ipomoea aquatica and Jussiaea repens administered orally at a dose of 1 teaspoon thrice daily for 1 week by the Chakma community of Tripura State, India, as hepatoprotective [120]; 1/2 cup of leaf infusion orally taken as remedy against gonorrhea by tribals of Mayurbhanj district of North Orissa, India [163]; stem used against ulcer, gastric, and whole plant against constipation by different tribes of Cachar district, Assam, India [164]; extract obtained from boiled plants used as antidiabetic by the Meitei-Pangal community of Thoubal district of Manipur, northeast India [165]; one teaspoon leaf juice mixed with equal amounts of *Centella asiatica* and cucumber juice orally taken against hypertension and excess bile secretion by the Tripuri and Reang tribes of Tripura State, India [166]; leaves used for headache, eye diseases, hookworm infection, and bile disorder by inhabitants of Similipal Biosphere Reserve, Orissa, India [167]; leaf and stem juice taken orally before meals as treatment for diabetes by the Marakh sect of the Garo tribe in Mymensingh district, Bangladesh [168]; tender shoots orally taken as a laxative by ethnic communities in Tinsukia district, Assam, India [60]; whole plants cooked and eaten as vegetable against edema in any part of the body by the Garo community of Tangail district, Bangladesh [169]; plant juice used against gonorrhea; leaf juice applied topically for prickly heats, and leaf juice orally taken against spermatorrhea by tea garden tribes of Darrang and Udalguri districts, Assam, India [170].	Central nervous system depressant activity [171], hepatoprotective [172], antioxidant [173].

17	*Ficus hispida* L.	Exudate from roots taken orally by ethnic groups in Disoi Valley Reserve Forest of Jorhat district, Assam, India, against diabetes, and curry prepared from leaf is taken in jaundice [67]; fruits used as hepatoprotective by some ethnic communities of Tripura State, India [120]; 50 g dried stem bark is boiled in water with 100 g dried stem and root bark of Ficus benghalensis; the decoction is taken once a day for a period of 6 weeks against diabetes by the Palliyar tribals in Sirumalai Hills, Western Ghats, Tamil Nadu, India [68]; fruits and bark used against leprosy, for blood purification, and for increasing lactation by Kani tribals of Agasthiyarmalai Biosphere Reserve, southern Western Ghats, India [174]; leaves and seeds used by Kavirajes of Chalna area, Khulna district, against diuretic, vomiting, and dermatitis [175]; fruits orally taken as anxiolytic in Natore and Rajshahi districts, Bangladesh [100]; stem used for cure of wounds by tribals in Buldhana district, India [176]; paste of fruits rubbed by tribal communities to treat headache in Jalgaon district, North Maharashtra, India [177]; leaves used against ringworm by the tribes of Paderu Mandalam, Visakhapatnam district, Andhra Pradesh, India [114].	Antineoplastic, cardioprotective, neuroprotective, anti-inflammatory [178].

18	*Glinus oppositifolius* (L.) A. DC.	Whole plant paste applied topically against skin diseases by traditional healers of South Orissa, India [179]; used against gastrointestinal disorders in Ashuganj of Brahmanbaria district, Bangladesh (plant part used not mentioned) [64]; whole plant juice used in Noakhali district, Bangladesh for improvement of appetite and as digestive aid; whole plant juice along with castor oil is applied to ears to cure ear ache; whole plant juice applied topically for itch, and skin diseases [59]; leaves are cooked and eaten for keeping the body cool in Pirojpur district, Bangladesh [180]; extract or curry of fresh leaves taken orally against skin diseases; leaf extract applied topically on wounds by inhabitants of three districts of West Bengal, India [181].	Antioxidant, hepatoprotective, immunomodulatory, antiprotozoal [182], antioxidant, antihyperglycemic [183].

19	*Ipomoea aquatica* Forssk.	Fresh leaf paste is applied on wounds and boils by the Yanadi tribe of Sriharikota Island, Andhra Pradesh, India [184]; fried leaves are orally taken for head reeling; leaf juice along with cow “ghee” (clarified butter) is taken for gonorrhea; leaf juice is taken as blood purifier and purgative in South Orissa, India [179]; crude extract of leaves applied to wounds and boils by the Chorei tribes of Southern Assam, North Eastern India [162]; juice obtained from macerated whole plant is orally taken as antidote to poisoning and against chicken pox in Kurigram district, Bangladesh [87]; whole plant used in digestive problems and liver diseases by rural people of “Chatara” block of Sonebhadra district, Uttar Pradesh, India [185]; leaves are orally taken for leucorrhea and to increase lactation in nursing mothers in Shitol Para village, Jhalokati district, Bangladesh [148]; used against gastrointestinal disorders in Iloilo, Philippines (plant part used not mentioned) [103]; leaf juice used in jaundice, urinary trouble, and nervous hindrance by the Nath people of Assam, India [76]; tender shoots used in diabetes and as galactagogue by ethnic communities of Tinsukia district, Assam, India [60].	Antidiabetic, antioxidant, anticancer, anti-inflammatory, antiarthritic, antimicrobial, antiulcer, nootropic, antiepileptic, central nervous system depressant, anxiolytic, hypolipidemic, diuretic, analgesic, antiscorpion venom [186].

20	*Ipomoea batatas* (L.) Lam.	Leaves are orally taken as blood tonic and leaves are mixed with salt to treat whitlow in Nigeria [187]; leaves are topically applied against boils by the Bench ethnic group of Ethiopia [188]; tubers used by tribals in Chitteri Hills, India, to treat diabetes [110]; leaves used for treating gingivitis and toothache in animals in Shitol Para village, Jhalokati district, Bangladesh [148]; roots used as aphrodisiac by tribes of Lalganj block of Mirzapur district, Uttar Pradesh, India [88]; used as digestive (tender leaves eaten boiled) by the Karbi tribe of Anglong district, Assam, India [189]; boiled tubers with skin on are orally taken for kidney problems in Oyo State, Nigeria [190]; leaves orally taken against diabetes in Yoruba medicine of south western Nigeria [191].	Antioxidant, antidiabetic, wound healing, antiulcer, antibacterial, antimutagenic [192].

21	*Leucas aspera* (Willd.) Link	Leaves used against gastritis in Sialkot district, Pakistan [66]; paste of plant used against pain and inflammation; decoction of plant orally taken with 1-2 seeds of *Syzygium aromaticum* for chronic phlegmatic fever in northern part of Nara Desert, Pakistan [135]; leaf and twig juice taken orally by the Chakma tribe of Tripura State, India, against childhood jaundice and liver cirrhosis [120]; plant extract taken orally with plant extract of *Phyllanthus amarus* and boiled leaves of *Eclipta prostrata* and buttermilk twice a day for a period of one week against jaundice by the Palliyar tribes of India [68]; leaves used against gastritis in Jalalpur Jattan, Gujrat district, Pakistan [136]; leaves boiled in water and the vapor inhaled to cure headache and fever by traditional healers of Kancheepuram district, Tamil Nadu, India [193]; leaf juice mixed with common salt taken orally by Kani tribals in India to cure indigestion in children [127]; whole plant are boiled in mustard oil and topically applied for treatment of severe pain in Faridpur and Rajbari districts, Bangladesh [194]; leaves and flowers used for treatment of colic in Greater Khulna Division, Bangladesh [195], macerated root is orally taken with table salt for excessive menstrual bleeding by the Tongchongya tribal community of Roangchaari in Bandarban district, Bangladesh [196]; leaves are rubbed over scorpion bitten area in Nagapattinam district, Tamil Nadu, India [197]; leaf juice mixed with water is orally taken against scabies; root juice mixed with goat's milk is taken three times a day for four days to cure poisonous bites by villagers in Kumaragiri Hills, Salem district, Tamil Nadu, India [198].	Antifungal, anti-inflammatory, analgesic, antioxidant [199].

22	*Malva verticillata* L.	Roots used by local inhabitants against urinary complaints in Kedarnath Wildlife Sanctuary in Western Himalaya, India [200]; dried and powdered roots used against dandruff, febrile illness, and headache by local inhabitants Bale Mountains National Park, Southeastern Ethiopia [201]; root decoction is orally taken against urinary tract infection by the Bhotia tribal community of in Indian Central Himalaya region [202]; leaves are eaten as vegetable against stomach ailments by ethnic communities of Tinsukia district, Assam, India [60].	Antidiabetic [203].

23	*Marsilea minuta* L.	Leaf juice taken with curd for insomnia and leaves fried in “ghee” (clarified butter) taken orally for epilepsy by rural people of Jajpur district, Odisha, India [73]; dried and powdered leaves taken with hot water in case of diabetes by the Valayian tribal people of Alagarkoil Hills of Madurai district, Tamil Nadu, India [204]; whole plant used by tribals against body ache in Jharkand, India [205]; whole plant used in coughs, spastic conditions of leg muscles, insomnia, and as sedative by local and tribal people of Kumaun Himalaya, Uttarakhand, India [206]; decoction of leaves taken with ginger to cure cough and bronchitis by village people of Rajasthan, India [207]; whole plants used in cough and spastic condition of leg muscle; whole plant paste taken with curd prepared from black cow's milk for epilepsy and leaf juice dropped in nostrils of nose for cure of migraine by tribals of Similipal Biosphere Reserve, Orissa, India [155]; leaves used against diabetes by Irula tribe of Kalavai village, Vellore district, Tamil Nadu, India [74]; whole plant juice taken orally against gastrointestinal disorders by a Christian community residing in Mirzapur village of Dinajpur district, Bangladesh [208]; fresh leaves and petiole juice used against migraine by tribals of Hadoti plateau, southeastern Rajasthan, India [209].	Hepatoprotective [210], antistress [211], antitussive, expectorant [212], antidiabetic [213], antiaggressive [214], antitumor [215].

24	*Moringa oleifera* Lam.	Leaves taken orally to reduce body heat; flowers advised to be taken as food to increase sperm production in men and to treat indigestion and eye diseases by traditional healers in Kancheepuram district of Tamil Nadu, India [193]; decoction of leaves, barks, seeds, and roots used for treatment of skin diseases, headache, rheumatism, and inflammation and as a detoxifying agent by villagers around Kimboza forest reserve in Morogoro, Tanzania [216]; seed powder taken with a glass of lukewarm water against indigestion and flatulence in North Bengal, India [150]; fresh leaf juice orally taken against menstrual pain in rural areas of Kerala, India [217]; bark used for fever and fits; leaves used against constipation; flowers used against coughs and male sterility and fruits used against infertility in men and women in Tamil Nadu, India [101]; fresh juice of root bark used against dental caries in Coastal Dakshina Kannada, India [218]; leaf, flower, and bark used against stomach pain and to increase fertility by Kani tribals of Pechiparai Hills, Tamil Nadu, India [127]; decoction of bark along with barks of *Alstonia scholaris*, *Mangifera indica* and *Aegle marmelos* used for treatment of jaundice by folk medicinal practitioners in Bangladesh [219]; seeds used for treatment of epilepsy by folk medicinal practitioners of Brahmanbaria, Narsinghdi, and Rajshahi districts of Bangladesh [220]; bark decoction taken orally against puerperal fever, pain, jaundice, and debility in villages of Sylhet district, Bangladesh [221]; stems are taken orally against rheumatism; flowers are cooked like vegetable and eaten as treatment for chicken pox by the Pahan tribe of Natore district, Bangladesh [222]; leaf juice taken orally against diabetes by the Garo tribal community of Netrakona district, Bangladesh [63].	Anti-inflammatory, antioxidant, antimicrobial, antihyperlipidemic, antifertility, anticancer, hepatoprotective, antiulcer, central nervous system depressant [223].

25	*Musa paradisiaca* L.	Root tincture is used against weak erection; stem juice taken orally for low sperm count, and two roasted unripe fruits taken orally daily as an aphrodisiac by the IFA Nkari people of Akwa Ibom State, Nigeria [224]; juice extract of leaf sheath used against snake venom by the Kani tribes of Agasthiyarmalai Biosphere reserve, India [174]; fruits taken orally with leaf and stem juice of *Basella rubra* and sugar to prevent excessive bleeding following childbirth in Kurigram district, Bangladesh [87]; fruits used for treating diarrhea and dysentery by the Zou tribe of Churachandpur district, Manipur, India [225]; ripe fruit taken orally with “lightning bugs” to enhance female fertility in Dhemaji district, Assam, India [226].	Antidiarrheal, antiulcer, antimicrobial, antihypertensive, hypoglycemic, hypocholesterolemic, antioxidant, antiallergic, antisnake venom [227].

26	*Musa sapientum* L.	Root tincture taken orally against weak erection and as an aphrodisiac by the IFA Nkari people of Nigeria [224]; leaves steeped in hot water and taken orally thrice daily for one week by the Kanuri tribe of northeastern Nigeria to treat anemia, yellow fever, and malaria [228]; fruits used in Thailand as a laxative [229]; stem and leaves used for memory enhancement and antiaging in Sagamu, Nigeria [230]; inflorescence used against bad dreaming, bed wetting by children, insanity and unusual behavior, and headache by the Tai-Khamyangs of Assam, India [126]; leaf and stem juice taken orally against fever by villagers of Vasu Vihar village, Bogra district, Bangladesh [61]; leaf juice applied to ears for ear ache due to cold, and root juice taken orally for helminthiasis in Dhamrai, Bangladesh [231]; exudates of rotten root applied to wounds by the Igede people of Nigeria [232]; fruits with black pepper are taken orally for respiratory problems and flowers are used in diabetes and genital disorders, while plant is used dysentery, high blood pressure, and rheumatic pain in Sonebhadra district, Uttar Pradesh, India [185].	Antidiarrheal, antiulcer, antimicrobial, hypoglycemic, hypocholesterolemic, antioxidant, diuretic, wound healing, antiallergic [227].

27	*Nelumbo nucifera* Gaertn.	Rhizome extract used against dysentery in Buldhana district, Maharashtra, India [233]; paste of young leaves along with fruits of *Phyllanthus emblica* applied on forehead to get relief from headache; flower petal decoction is orally taken against diarrhea; young flower paste is used as a cardiotonic and for fever and liver ailments; dried seed powder taken with cow milk against headache; young seed paste applied topically for skin diseases; powdered root taken for ringworms; root paste taken in lemon juice for piles in South Orissa, India [179]; dried flower powder taken with ghee orally for treatment of piles by the Mullu kuruma tribe of Wayanad district, Kerala, India [109]; flower juice used by tribals in Chitteri Hills, India, to treat diabetes [110]; rhizomes used by tribals of Pedabayalu Mandalam, Andhra Pradesh, India, to treat dysentery [114]; tuber is eaten raw as treatment for gastrointestinal problems by villagers of Gingee Hills, Villupuram district, Tamil Nadu, India [234]; whole plant used in heart trouble, urinary diseases, bleeding piles, and as nerve tonic; seeds used during pregnancy and also used as diuretic, sedative, and expectorant (plant part used not mentioned) in Sonebhadra district, India [185]; seed powder is taken with honey for 40 days by Gond tribe of Adilabad district, Andhra Pradesh, India, for infertility [115]; decoction of red-flowered plant orally taken on an empty stomach once a day by tribals of Similipal Biosphere Reserve, Orissa, India, for treatment of blood dysentery [118].	Anti-ischemic, antioxidant, hepatoprotective, anti-inflammatory, antifertility, antiarrhythmic, antifibrosis, antiviral, antiproliferative, immunomodulatory (seeds), antidiarrheal, hypoglycemic, sedative, diuretic, anti-inflammatory, antioxidant, antipyretic, immunomodulatory (rhizome), hypoglycemic, antioxidant, aldose reductase inhibitory, antibacterial, aphrodisiac, antipyretic, antiplatelet (flower), cardioprotective, antiviral, antioxidant, lipolytic, hypocholesterolemic, antiobesity, hepatoprotective, anticancer (leaves) [235].

28	*Nymphaea pubescens* Willd.	Rhizome extract taken with sugar candy taken orally twice a day for 3 days against leucorrhea by tribes of Kinwat forest, Nanded district, Maharashtra, India [236]; paste of rhizomes and seeds of *Piper nigrum* applied externally on neck against goiter by tribals of Boudh district, Odisha, India [237]; decoction of rhizome of red-flowered plant used against blood dysentery; rhizome juice taken for leucorrhea; powdered rhizome with honey taken for piles, dysentery, and dyspepsia; root juice taken to keep stomach cool and get relief from burning sensations during urination; root paste of red-flowered plant taken for menorrhagia; root paste along with flowers of *Hibiscus rosa*-*sinensis*, bark of *Ficus religiosa*, and seeds of *Sesamum indicum* taken for abortion [179]; root is tied on waste of pregnant woman to prevent abortion by the Tharu tribe of Devipatan division, India [238]; roots used against dysentery by tribes of Pedabayalu Mandalam, Visakhapatnam district, India [114].	Hepatoprotective [239], anticancer [240].

29	*Oxalis corniculata* L.	Leaves used against diarrhea and dysentery in Gujrat district, Pakistan [136]; plant paste filtered and used as eye drop against eye diseases by ethnic groups of Disoi Valley Reserve Forest, India [67]; leaves used against dysentery by tribes of Mirzapur district, India [88]; leaf juice with curd is orally taken against diarrhea and dysentery by the Mullu kuruma tribe of Wayanad district, India [109]; used against stomach complaints, piles, colic, and dysentery in Manipur, India (plant part used not mentioned) [106]; extract of aerial vegetative portion taken with sugar against abdominal pain and diarrhea by the Tai Ahom tribe of Dibrugarh district, Assam, India [241]; whole plant used against oral ailments in Dharwad district, Karnataka, India [242]; leaf paste is applied over forehead to treat headache; leaves are consumed as salad for indigestion and loss of appetite by the Khamti tribe of Arunachal Pradesh, India [57]; stem, bark, and root used against dyspepsia, piles, anemia, and tympanitis by tribals of Kaptipada Forest Range, India [71]; whole plant juice is orally taken against dog bite and snake bite by the Khatriya and Kashya clans of the Bagdi people of Rajbari district, Bangladesh [243].	Antiinflammatory, anxiolytic, anticonvulsant, antifungal, antiulcer, antinociceptive, anticancer, antidiabetic, hepatoprotective, hypolipidemic, abortifacient, antimicrobial, wound healing, antidiarrheal, antiamebic, antiepileptic [244].

30	*Raphanus sativus* L.	Roots used against jaundice in Bangladesh [245]; used against whooping cough in Tunisia and Italy (plant part used not mentioned) [246]; leaves and roots orally taken against acidity in Firozabad district, India [247]; used against syphilis in Samahni Valley, Azad Kashmir, Pakistan [248]; used against coughs in Jalgaon district, India [249]; roots used against urinary trouble by tribes of Pedabayalu Mandalam, Visakhapatnam district, India [114]; roots are consumed to regularize digestive complaints in Buldhana district, India [233]; fresh leaf juice is orally taken with sugar candy and butter milk to cure piles in Dharmabad Taluka of Nanded district, India [250]; fresh roots or leaves are eaten raw against urinary complaints and as a diuretic by tribes of Northeast Gujarat, India [251]; seeds are taken orally against sexual debility by natives of Bargarh district, Orissa, India [252].	Antihypertensive, antiobesity, antidiabetic, constipation, cough [253].

31	*Saccharum spontaneum* L.	Whole plant used for improvement of appetite and treatment of abdominal pain in Gujrat district, Punjab, Pakistan [136]; pulp of crushed leaves used against pus formation in any part of the body in Jaunsar-bawar, Dehradun district, India [254]; crushed roots are boiled in water and orally taken against asthma in Mirzapur village of Dinajpur district, Bangladesh [208]; paste prepared from roots of the plant and roots of *Cynodon dactylon* taken with cow milk and sugar early in the morning for 1 month against leucorrhea in Meerut district, Uttar Pradesh, India [255]; used against gastrointestinal disorders in Iloilo, Philippines (plant part used not given) [103].	Antiurolithiasis [256], antioxidant [257].

32	*Scoparia dulcis* L.	Stem infusion is used by the ethnic communities of Tinsukia district, Assam, India against gastritis [60]; decoction of leaves taken continuously for a week for treatment of sore throat by Kol tribals of Similipal Bioreserve, Orissa, India [118]; leaves and twigs taken orally as a hepatoprotective agent (i.e., against jaundice) by the Darlong tribe of Tripura State, India [120]; crushed root extract orally taken for stomach pain, urinary disorders, and kidney stone by the Kurichya tribe of Kerala, India [124]; leaves, flowers, and fruits are ground into a paste and used against wounds and to control bleeding by the Kani tribals of Tamil Nadu, India [127]; used against diarrhea in Trinidad and Tobago (plant part used not mentioned) [258]; decoction of leaves of the plant along with leaves of *Eclipta alba* and *Cynodon dactylon* used against diabetes by the Santal tribe of Thakurgaon district, Bangladesh [259]; whole plant extract is orally taken against urinary diseases by the Kurichar tribe of Wayanad district, Southern Western Ghats, Kerala, India [260]; whole plant used against cough, bronchitis, and kidney trouble by people of Golaghat district, Assam, India [261]; leaf juice orally administered by the Pankho tribal community in Rangamati district, Bangladesh, against spermatorrhea [262]; leaf juice used by folk medicinal practitioners in Kurigram district, Bangladesh, against “meho” (diabetes) [87]; plant decoction used by the Darlong tribe of Tripura State, India, against jaundice [120]; paste of tender shoots along with *Paederia scandens* in the ratio of 2 : 1 taken 3 times per day for 2 days during menstruation against female infertility in Dhemaji district of Assam, India [226]; macerated leaves taken orally against fever by the Igede people of Nigeria [232]; 50 g whole plant together with 50 g whole plant of *Phyllanthus amarus* and 50 g *Sida acuta* (whole plant) are made into a paste and mixed with 250 mL drinking water and taken orally twice a day for 1-2 days to treat snake bite by tribes of South Surguja, Chhattisgarh, India [263]; leaf ground into a paste and used for wound healing by tribal people in Southern India [264]; leaf extract is orally administered by Meche people of Jhapa district, eastern Nepal, against continuous weeping by baby [265].	Antidiabetic, analgesic, anti-inflammatory, antiviral, antimalarial, neurotropic, anticancer, hypertensive, sedative, diuretic [266].

33	*Sesbania grandiflora* (L.) Pers.	Bark used in Visakhapatnam district, India, by tribal people against diarrhea [114]; leaves used against skin lice by rural people of Mayurbhanj district, Orissa, India [163]; leaves prepared in the form of soup and taken orally by the Valaiyan tribe of Alagarkoil Hills, Madurai district, Tamil Nadu, India, as vermifuge and against peptic ulcer [204]; 50 mL of leaf decoction taken orally on an empty stomach as vermifuge and against stomach ailments by tribal and rural people of Sirumalai Hills, Dindigul district, Tamil Nadu, India [267]; leaf, young fruit and bark used against headache and fever by the Malayaraya tribe of Vannapuram village in Idukki, Kerala, India [268]; cooked flowers taken orally against dizziness in Mysore and Coorg districts, Karnataka, India [269]; leaf decoction taken orally for body cooling by Kani tribals of Tirunelveli hills, Western Ghats, India [270]; cooked leaves are orally taken to give cooling effect to infected eyes by the Irula and the Soliga tribe of Sathyamangalam forests of Erode district, Tamil Nadu, India [271].	Wound healing, antimicrobial, hypolipidemic, antiulcer, anti-inflammatory, antiarthritic, antioxidant, antihelmintic, antidiarrheal, analgesic, diuretic, central nervous system depressant, laxative [272].

34	*Spilanthes paniculata* Wall. ex DC.	Used against toothache by Tripura tribal medicinal practitioners in Tripura State, India [82]; leaf juice is orally taken (1 teaspoon thrice daily for 3-4 days) against jaundice and cirrhosis by the Halam tribe of Tripura State, India [120]; flower heads are chewed against toothache in Coastal Dakshina Kannada, India [218]; flowers and fruits are directly chewed by the Adi tribes of Lower Dibang district of Arunachal Pradesh, India, for toothache [273]; leaves are used against diarrhea, dysentery, and high blood pressure by the Naga and Kuki tribes of Senapati district, Manipur State, India [274]; leaves are orally taken by the Apatani tribe of Arunachal Pradesh, India, against constipation [275].	Antifungal, antipyretic, local anaesthetic, bioinsecticide, anticonvulsant, antioxidant, aphrodisiac, analgesic, pancreatic lipase inhibitor, antimicrobial, antinociceptive, diuretic, vasorelaxant, anti-human immunodeficiency virus, toothache relieve, anti-inflammatory [276].
